# Primary care in five European countries: A citizens’ perspective on the quality of care for children

**DOI:** 10.1371/journal.pone.0224550

**Published:** 2019-11-11

**Authors:** Janine A. van Til, Catharina G. M. Groothuis-Oudshoorn, Eline Vlasblom, Paul L. Kocken, Magda M. Boere-Boonekamp

**Affiliations:** 1 University of Twente, Technical Medical Centre, Department of Health Technology and Services Research, Faculty of Behavioural, Management and Social Sciences, the Netherlands; 2 TNO, Department of Child Health, Leiden, The Netherlands; 3 Public Health and Primary Care Department of LUMC, Leiden, The Netherlands; Pomeranian Medical University in Szczecin, POLAND

## Abstract

**Objective:**

As part of the Models of Child Health Appraised (MOCHA) project, this study aimed to answer the following research questions: 1) How do European citizens perceive the quality of primary health care provided for children? And 2) What are their priorities with respect to quality assessment of primary health care aimed at satisfying children’s needs?

**Methods:**

Nine potential attributes of quality of primary care were operationalized in 40 quality aspects. An online survey was used to elicit opinions in a representative sample of citizens of Germany, the Netherlands, Poland, Spain, and the United Kingdom. Data collection comprised: background characteristics; perceived quality of primary health care for children; and priority setting of quality aspects. Descriptive analysis was performed and differences between groups were tested using Chi-Square test and ANOVA.

**Results:**

Valid results were obtained from 2403 respondents. Mean satisfaction with quality of primary care ranged from 5.5 (Poland) to 7.2 (Spain). On average, between 56% (Poland) and 70% (Netherlands) of respondents had a positive perception of the primary health care system for children in their country. The ability of a child to limit their parents’ access to the child’s medical records was judged most negatively in all countries (average agreement score 28%, range 12–36%). The right of a child to a confidential consultation was judged most differently between countries (average agreement score 61%, range 40–75%). Overall top-10 priorities in ensuring high quality primary care were: timeliness (accessibility); skills/competences, management, facilities (appropriateness); no costs (affordability); information, dignity/respect (continuity); and swift referrals, collaboration (coordination).

**Discussion:**

Between countries, significant differences exist in the perceived quality of primary care and priorities with regard to quality assessment. Taking into account the citizens’ perspective in decision-making means that aspects with low perceived quality that are highly prioritized warrant further action.

## Introduction

The health of children is of utmost importance to themselves and their families and as future workers, parents and carers in society. The individual and population health of adults as well as children is determined by a range of factors, such as genetics, biology, social factors, individual behavior, policymaking and healthcare services [1, 2]. It is the interrelationships among these factors that determine health.

Healthcare services are structured differently throughout the European Union, and there is little research into what works best. For all age groups, primary care is the first level of professional care service, where people present their health problems, and where the majority of the population’s curative and preventive health needs can be satisfied [3]. Based on a literature review, Kringos et al. identified three dimensions of primary care structure: primary care governance, economic conditions of primary care, and primary care workforce development; and four dimensions related to the primary care process: accessibility, continuity, coordination and comprehensiveness [3, 4]. Primary care services: 1) should be easily available close to where people live with no obstacles to access at the time of need; 2) should provide continuity of care over the life course; 3) should coordinate the different teams of health professionals that collaborate in the care for a certain patient; and 4) should feature preventive care throughout the life course, including prevention of disease or health problems, lifestyle advice, and physical and mental illness prevention [5–7]. Variation in the overall strength of primary care in Europe appears to refer to different degrees in which the above-mentioned dimensions of primary care structure and process have been developed in countries [4]. Primary care for children fits within this generic picture, but has to accommodate the physiological and intellectual development of the child; the range of specific childhood conditions and illnesses; the high dependence, at least in the early years, on parents who are not the ‘patient’ but vital to the child’s wellbeing and their health care access; and the complex interaction with society including in particular the education sector.

Decision makers increasingly use the strengthening of primary care in their strategy to cope with the challenges that healthcare systems are facing like ageing populations, health inequities, more complex needs, technological changes, etc. [4]. One of the aspects relevant to decision makers is how citizens who pay and/or make use of healthcare services appraise the quality of the primary care. This knowledge can help policy makers as well as primary care professionals to set priorities in their effort to make health care delivery more responsive to the citizen’s needs [8, 9]. The available literature on citizens’ views on primary care for children is mainly focused on measuring patient experiences of quality of healthcare, usually for specific age groups (e.g., adolescents and young adults), patients with chronical conditions (e.g., cancer, cystic fibrosis), specific domains (e.g., effectiveness, patient-centeredness, care coordination) and specific dimensions of health services (e.g., the transition process from child to adult services) [10–17]. In 2017, Kleij et al. published a systematic review on the aspects of primary care that have been included in previous preference studies and which of them were the most preferred aspects [8]. None of the 18 included studies focused on what citizens generally find important characteristics of good-quality primary care for children.

In the MOCHA project (Models of Child Health Appraised), a systematic, scientific evaluation of the types of primary care services for children that exist in Europe, was performed (http://www.childhealthservicemodels.eu/). The project comprised of a multidisciplinary study of appraisal of primary care for children and young people in 30 EU/EEA countries. Topics included are e.g., the visibility of children in data and policy systems; challenges in measuring quality and outcome of care; stakeholders’ views on primary care delivery; vulnerable children and ensuring equity of care; interfaces of models of primary care with secondary, social and complex care; models of effective school and adolescent health services; health workforce in child health; the role of E-Health. The aim of the MOCHA-project was to identify optimal, sustainable and cost-efficient child-centred and prevention oriented primary child healthcare models. As part of the MOCHA-project we aimed to elicit formative values from citizens of European countries and to determine citizen priorities in the assessment of the quality of a child-oriented primary care system. Research questions were: 1) How do European citizens perceive the quality of primary health care provided for children? And 2) What are their priorities with respect to quality assessment of primary health care aimed at satisfying children’s needs?

## Methods

To elicit the citizens’ perspective a digital questionnaire, the Priorities fOr Child Health Care Assessed (POCHA) instrument was developed. The developmental process, target population and recruitment, and data analysis are described below.

### Target population and recruitment

The aim of the study was to get a broad perspective on the perceived quality of primary health care for children and priorities of EU citizens for assessment of the quality of primary health care in different countries. Within the time and financial limitations to this study, we were not able to recruit in all EU countries. Given the wide variety of health care systems within Europe [18], we selected countries which differed on main characteristics of the health care system which might influence the experience of citizens in the countries. These characteristics are: type of lead practitioner, gatekeeping, and the organizational place of preventive care services. Germany, the Netherlands, Poland, Spain and the United Kingdom were selected. Characteristics of these countries are presented in Table 1.

**Table 1:** Characteristics of participating countries.

	GERMANY	NETHERLANDS	POLAND	SPAIN	UNITED KINGDOM
**PRIMARY CARE LEAD PRACTITIONER**	Primary care paediatrician	General practitioner	Combined (general practitioner /paediatrician)	Primary care paediatrician	General practitioner
**REFERRAL / ACCESS SYSTEM TO SECONDARY CARE**	Open access	Primary care is gatekeeper to other health services	Primary care is gatekeeper to other health services	Primary care is gatekeeper to other services/health care levels	Primary care is gatekeeper to other health services
**ORGANIZATIONAL PLACE OF PREVENTIVE CARE FOR CHILDREN**	Services are partly integrated in primary care and partly separated	Services have a separate lead; the preventive child physician	Services are integrated in primary care.	Services are partly integrated in primary care and partly separated	Services are partly integrated and partly separated

The target respondent population were citizens of the five EU countries. We aimed to get the perspective of the working and tax paying part of the country population. Therefore, we recruited men and women, parents and non-parents and we chose to limit age of the respondents to between 18–65 years of age.

We used best worst scaling to elicit priorities in respondents. Unfortunately, no power calculation methods are available for Best Worst Scaling [19]. The sample size was determined at 500 because this enabled us to recruit sufficient numbers of respondents in all age groups and of both genders to perform the required analysis. It was decided to recruit equal numbers of respondents in the five countries, despite differences in country population, because then between country comparison is not influenced by differences in observations between countries. To be able to compensate for dropout, we recruited about 30 respondents extra in all five countries. The sample was recruited by Research Now SSI in February 2018, through the dynamic sampling platform Dynamix^™^, using quota for background characteristics gender, age and location, based on these characteristics’ distribution in each of the five countries. This platform recruits participants via partnerships with trusted loyalty programs as well as via banner ads, pop ups and messages on websites, TV advertising and offline (closed survey). The platform uses a reward strategy to achieve maximum representation within online sample, however, participation is voluntary. In addition, a suite of controls is used to prevent duplicates in the online samples and to ensure the quality of survey data (digital fingerprinting, address verification against USPS databases and third-party verification).

### Questionnaire development

#### Attributes of the quality of a child-oriented primary care system

As there was no validated instrument available to assess the citizen’s experiences and perceptions of the quality of a child-oriented primary care system and to determine citizen priorities, a new questionnaire was developed [20]. Based on the model developed by the MOCHA working group [18] and other literature sources [5, 21–26], nine potential attributes of a primary care system from a child, youth and carer centred perspective were identified and defined: accessible, affordable, appropriate, confidential, continuous, coordinated, empowering, equable, and transparent (Table 2). Subsequently, these attributes were operationalized in 40 different aspects to cover the full definition of each attribute (S1 Table).

**Table 2:** List of attributes of a child-oriented healthcare system from a child, youth and carer centred perspective.

Attribute	Definition
Accessible	Accessible primary care is available within reasonable reach of parents and children, with ample opening hours, good appointment systems and other aspects of service organization and delivery that allow children to obtain the services when they need them (adapted from Evans et al. 2013 [22]).
Affordable	Affordable primary care can be accessed without inordinate financial barriers, such as high co-payments or cost-sharing arrangements (adapted from Kringos et al. 2010 [5]).
Appropriate	Appropriate primary care is effective in meeting the child’s needs, timely and of high technical quality (adapted from Levesque et al. 2013 [21]).
Confidential	Confidentiality in primary care is the right of a child to have personal, identifiable medical information kept private if they choose to, from medical professionals as well as parents (developed in the project).
Continuous	Continuous primary care is the experience of a continuous caring relationship with the health care professional(s) by a single child and its parents over time, that is responsive of the child’s changing needs (based on Kringos et al. 2010 [5], Haggerty et al. 2003 [23], and Price et al. 2013 [24]).
Coordinated	Coordinated primary care is deliberately organizing child care activities and sharing of information among all of the participants concerned with a child’s care with the aim to achieve safer and more effective care (McDonald et al. 2014 [25]).
Empowerment	Empowerment in primary care is a process through which children and parents gain greater control over decisions and actions affecting a child’s health (WHO definition [26]).
Equable	Equable primary care is the absence of systematic and potentially remediable differences in access to primary care and health status across population groups (adapted from Kringos et al. 2010 [5]).
Transparent	Transparent primary care is the degree to which a healthcare service or provider is open to children and parents about their quality, cost structure, services and work method (Levesque et al. 2013 [21]).

#### Questionnaire

The POCHA questionnaire consisted of the following parts:

1. Background characteristics: age (in years: ≤ 19, 20–24, 25–29, ……, 65–69, ≥ 70); gender (female, male); country (Germany, Netherlands, Poland, Spain, United Kingdom) and the countries’ region; number of children (1, 2, 3, other); children < 18 (yes/no); highest level of completed education (low, middle, high; country-specific classification [19]); size of the city, where the respondent lives (< 100, 100–999, 1 000–9999, 10 000–19999, 20 000–99999, 100 000–199999, 200 000–1 000 000, > 1 000 000); health status (respondents were asked whether their child(ren) had a medical condition that lasted longer than 6 weeks and, if yes, they could select from a list of conditions retrieved from the National Institute for Public Health and the Environment (RIVM) in the Netherlands[27]: Eczema, Asthma, Hay fever, Allergy, Stomach ache, Headache, Back problems, Fatigue, Sleep problems, Depressive complaints, Hyperactivity and ADHD, Constipation, Overweight and obesity, Other:..,); and current healthcare use of any child(ren) < the age of 18;
2. Assessment of the respondents’ perceived quality of the primary care system, by measuring: 1) overall satisfaction score, on a scale of 1 (very dissatisfied) to 10 (perfectly satisfied); and 2) experiences (parent-respondents) or perceptions (other respondents), through questions to respondents where they had to rate their agreement with statements on quality aspects (n = 10) on a 5-point rating scale (strongly disagree to strongly agree);
3. Respondents’ prioritization of the 40 aspects of quality of the primary care system. Because it is unfeasible for a respondent to rank 40 items, the partial ranking technique of best-worst scaling (BWS) was used [28]. Eight different sets of ten BWS questions were generated, each containing the 40 items in different combinations from each other (S1 Table). Each respondent received two out of these eight sets of ten questions (randomly selected). In each question, four items (quality aspects) were presented and respondents were asked to select the most and least important item in that set. Thus, each item could be presented twice to each respondent (4 items per question*20 questions)/ total of 40 items). An experimental design was generated in R-software to vary the combinations of items over the questions to ensure there was no overlap (item combinations shown multiple times).

The English questionnaire was translated in Dutch, German, Spanish, and Polish, by certified translators. Translated questionnaires were checked by two to three native speakers who were familiar with the country’s primary care system. Before fielding the questionnaire in February 2019, the usability and technical functionality of the electronic questionnaire were tested by the market agency responsible for recruitment and pilot testing in 10 respondents.

### Ethics

According to the criteria of the Dutch Medical Research Involving Human Subjects Act, this study did not need to be submitted for ethical approval by a Medical Ethical Committee. The study was reviewed and approved by the ethical committee of the Faculty of Behavioural, Management and Social Sciences of the University of Twente under file number BCE17583, on September 19, 2017. The purpose of the study, the length of filling in the questionnaire, and the investigators were mentioned at the beginning of the questionnaire. No personal information was collected or stored.

### Data-analysis

Respondents’ background characteristics and experiences/perceptions of quality of primary care were analyzed using descriptive statistics. Average and standard deviation of the satisfaction score (number between 1 and 10) were calculated per country and over all countries. Perceived quality of care (rated on a 5-point scale) was collapsed into negative perception (score 1 and 2), neutral (score 3) and positive perception (score 4 and 5). The frequency (%) of respondents with positive perception was presented per country and overall per item as the quality score. Also, the average quality score was calculated over all items for each country, resulting in an overall perceived quality of the healthcare system score per country. Chi-square tests were performed to test whether there were significant differences in background characteristics and perceived quality of care between respondents from the different countries.

Priorities for aspects of quality of care were calculated using counts analysis on an individual and a group level. First, the number of times each item was selected as best (best count) and worst (worst count) was counted at individual level. As each item was shown twice to each respondent, the maximum number of times an item could be selected as most and least important was two. Best-worst scores were calculated by subtracting worst count from best count. Individual best-worst counts ranged from– 2 (not important) to +2 (important). Group priorities were calculated by summing the best and worst counts for each individual in the group for each item. Best-worst scores were normalized over groups by dividing the best-worst count by the number of times each item was presented to the group (2*number of respondents in the group) and multiplying this ratio with 100, resulting in a possible priority score ranging from +100 to -100. Group best-worst scores were calculated for each country. Priority scores were compared between countries with ANOVA (F-test). Separate analyses were performed for parents of children (< 18 and ≥ 18) and non-parents within the countries.

## Results

### Background characteristics

In total, 3375 respondents started the survey, of which 2640 respondents completed. Of these, 237 were excluded because of speeding or nonsensical answers in the qualitative questions [results not presented]. Dropout rates were between 10.8% and 6.5% (S1 Appendix). In total, 2403 respondents were included in the analysis, of which 469 were respondents from Germany, 469 from the Netherlands, 478 from Poland, 491 from Spain, and 496 from the United Kingdom. The sample was representative of the population in the country in terms of age, gender and physical location of citizens between 18 and 65 years of age. Due to the sampling methodology used in this study, a response rate could not be calculated. The background characteristics of respondents are presented in Table 3.

**Table 3:** Background characteristics of the 2403 respondents.

Background Characteristics	Germany		The Netherlands		Poland		Spain		United Kingdom		F-test	p-value
	n	(%)	n	(%)	n	(%)	n	(%)	n	(%)		
**Age**											1.5	0.190
19 years or younger	12	(3)	17	(4)	9	(2)	7	(1)	16	(3)		
between 20 and 29	82	(17)	75	(16)	108	(23)	88	(18)	97	(20)		
between 30 and 39	95	(20)	87	(19)	102	(21)	115	(23)	110	(22)		
between 40 and 49	101	(22)	112	(24)	95	(20)	129	(26)	112	(23)		
between 50 and 59	125	(27)	112	(24)	106	(22)	116	(24)	103	(21)		
between 60 and 69	54	(12)	66	(14)	58	(12)	36	(7)	58	(12)		
**Gender**											1.8	0.121
Female	247	(53)	219	(47)	261	(55)	259	(53)	266	(54)		
Male	222	(47)	250	(53)	217	(45)	232	(47)	230	(46)		
**Children**											30.8	0.000
No Children	222	(47)	195	(42)	162	(34)	179	(36)	214	(43)		
Children < 18	143	(30)	148	(32)	173	(36)	235	(48)	173	(35)		
Children ≥ 18	104	(22)	126	(27)	143	(30)	77	(16)	109	(22)		
**City size**											16.1	0.000
1–20 000	169	(36)	186	(38)	116	(24)	189	(40)	839	(35)		
20 000–100 000	147	(31)	122	(25)	112	(23)	111	(24)	595	(25)		
100 000–200 000	76	(16)	54	(11)	65	(13)	37	(8)	281	(12)		
200 000–1 000 000	59	(13)	65	(13)	107	(22)	91	(19)	429	(18)		
> 1 000 000	18	(4)	69	(14)	91	(19)	41	(9)	259	(11)		
**Educational level**												
Low	143	(31)	87	(19)	12	(3)	54	(11)	140	(28)		
Middle	216	(46)	198	(42)	239	(50)	168	(34)	180	(36)		
High	110	(23)	184	(39)	227	(48)	269	(55)	176	(36)		

The age and gender distribution of the citizen samples of the five participating countries were comparable. The family composition differed significantly between countries; the percentage of respondents with children below 18 years of age in the samples ranged from 30% (Germany) to 48% (Spain)(p<0,000). The distribution of the size of the city, where the respondents live, also differed significantly between the countries (p<0.000). The distribution of the educational level of the respondents could not be compared between countries, because of the difference in categorization of the educational systems.

Of the respondents with child(ren) below the age of 18, 22.5% (196/872) indicated that at least one child had a health problem that lasted more than 6 weeks in the past year. This percentage varied per country: 17% in Germany, 24% in the Netherlands, 24% in Poland, 20% in Spain, and 27% in the United Kingdom. The conditions that were reported most frequently were allergy-related conditions, i.e. unspecified allergy, asthma, eczema and hay fever. The majority of respondents (67%) reported one condition in their child(ren), around 14% reported two and another 18% reported three or more conditions in their child(ren).

The average number of contacts of respondents with children below 18 years of age (n = 872) with healthcare professionals in the past 12 months did not differ much between countries (range 2.9–3.4). However, there were statistically significant differences between countries in the percentage of respondents who had contact with the GP (Germany 90%, Netherlands 84%, Poland 90%, Spain 93%, United Kingdom 84%), the district nurse (Germany 8%, Netherlands 11%, Poland 29%, Spain 15%, United Kingdom 27%), and the pediatrician (Germany 71%, Netherlands 34%, Poland 75%, Spain 78%, United Kingdom 29%). The percentage of respondents who had contact with other care providers did not differ significantly between countries; mean percentages for contacts with the dentist was 85% (range 77–93%), with the physiotherapist 23% (range 20–25%), with the social worker 13% (range 9–17%), and with a hospital specialist 36% (range 31–39%).

### Perceived quality of primary health care

Mean primary health care satisfaction score was 5.5 (SD = 2.2) for Poland, 6.9 (SD = 1.7) for Germany, 6.9 (SD = 1.3) for the Netherlands, 7.0 (SD = 1.7) for the United Kingdom, and 7.2 (SD = 1.6) for Spain.

**Fig 1:**
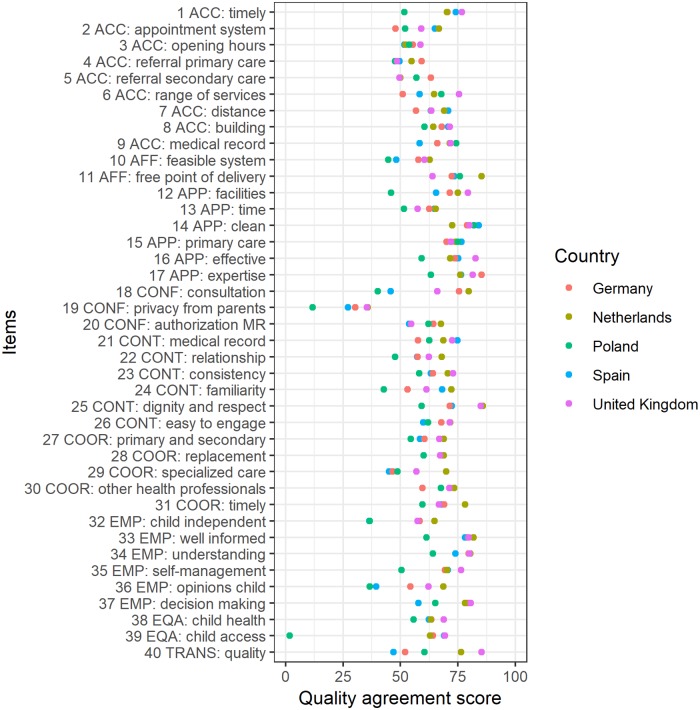
The perceived quality of the primary care system for children in five EU countries. Quality agreement scores are presented per country per quality aspect. For the full description of the 40 items, see S1 Table.

In Fig 1 the quality agreement scores are presented per country per quality aspect (see S1 Table for the list of quality aspects, item number and descriptions, and S2 Table for the percentages of agreement). Agreement with the quality aspects of primary care for children ranges from 12% (Poland: a child can limit his parents’ access to his medical record; item 19) to 86% (Netherlands: healthcare professionals show dignity and respect; item 25).

For some aspects, the perceived quality is very comparable in all countries, for instance for having ample opening hours of primary care services (item 3), provision of care in the primary care setting if possible (item 15) and the availability of replacement of care providers in case of sickness (item 28). For 27 out of 40 aspects of high-quality care, the perceived quality is significantly different between countries; the three items with the largest differences between countries are: the right of the child to a confidential consultation (item 18); transparency about the quality of care (item 40); and the availability of facilities and equipment in primary care (item 12). Items with low perceived quality (<60% agreement) in at least four out of five countries are: the extent to which a child can limit his parents’ access to the child’s medical records (item 19); the extent to which a child can express his opinions independently from his parents (item 32); the availability of specialized care within the primary care provider’s practice (item 29); and the possibility to make an appointment with other primary care providers (item 4). Items for which there was high agreement (>75% agreement) in at least four out of five countries are: the adequacy of the skills and competencies of care providers (item 17) and the extent to which children and their parents are well informed about (the management of) the child’s health (item 33).

Based on the average agreement over all 40 quality aspects, the overall quality agreement was highest in the Netherlands (70%), followed by the United Kingdom (68%). Overall quality agreement was lower in Germany (64%) and Spain (62%), and lowest in Poland (56%).

### Priorities in quality assessment of primary health care

**Fig 2:**
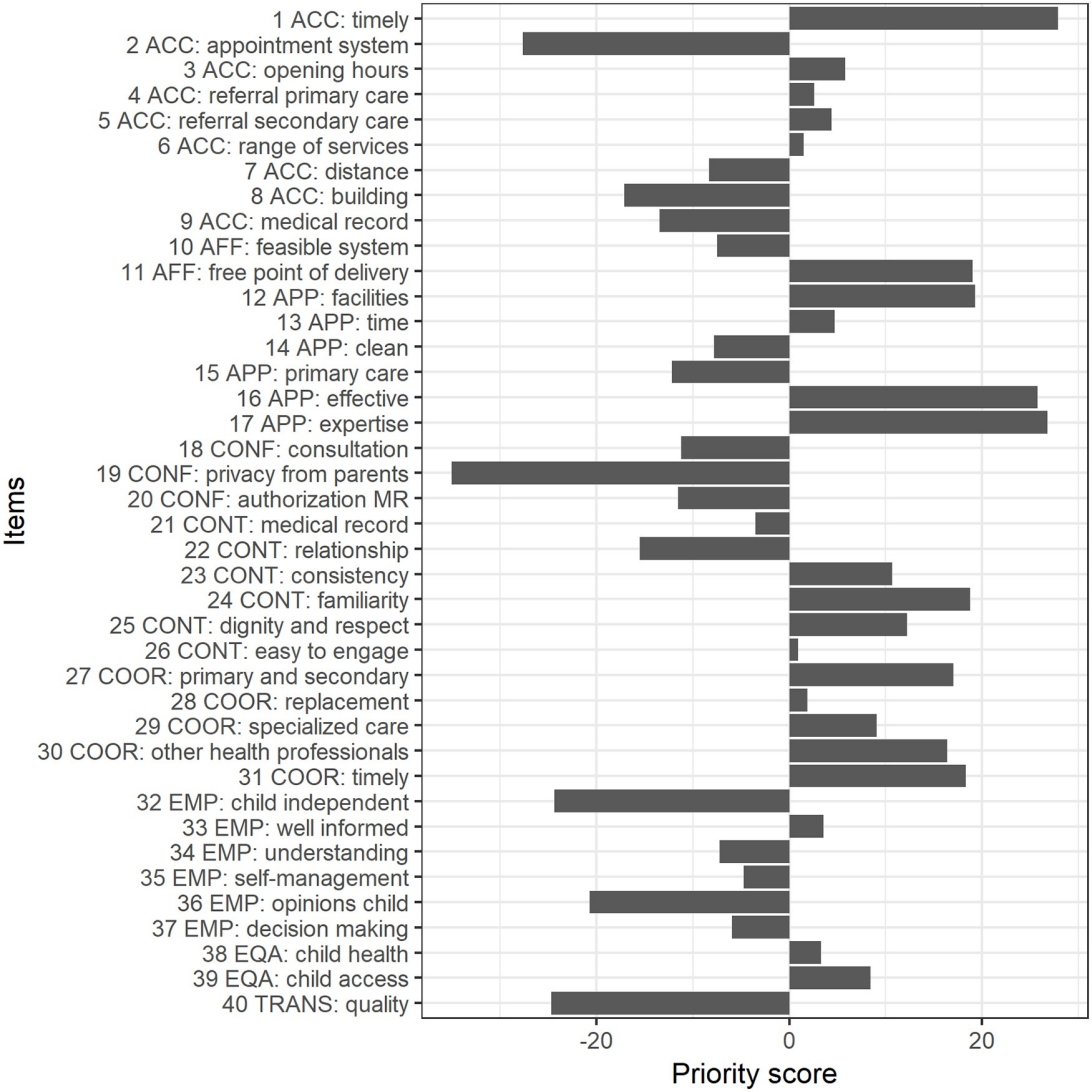
Overall priority scores for aspects of quality of primary care for children according to the respondents in five EU countries based on best-worst scaling. For description of the 40 items, see S1 Table.

Fig 2 shows the overall priority scores for aspects of quality of primary care for children according to the 2403 respondents in the five EU countries (group results). More positive scores indicate higher priority, while more negative scores indicate lower priority. All priorities are relative. The top ten of overall priorities were:

1. Primary care providers provide care within a reasonable amount of time, given the severity of the health issue (item 1)
2. Primary care providers have the skills and competences to provide the care a child needs (item 17)
3. In primary care, a child’s health problems are effectively managed (item 16)
4. In primary care, the facilities and equipment are available to deliver the services that are needed for children (item 12)
5. Primary care services for a child are free at the point of delivery, or out-of-pocket costs are fully covered or repaid (item 11)
6. All healthcare providers involved in the care of a child know about each other’s involvement, trust each other and work together (item 24)
7. Primary care providers treat children and their parents with dignity and respect (item 25)
8. In primary care, a child is referred to other healthcare providers swiftly if this is needed (item 31)
9. If a child needs specialised and long-term care, hospitals and primary care providers collaborate to offer care close to home (item 27)
10. If the main primary care provider of a child is not able to meet the needs of that child, that care can be given by other health professionals within primary care (item 30)

**Fig 3:**
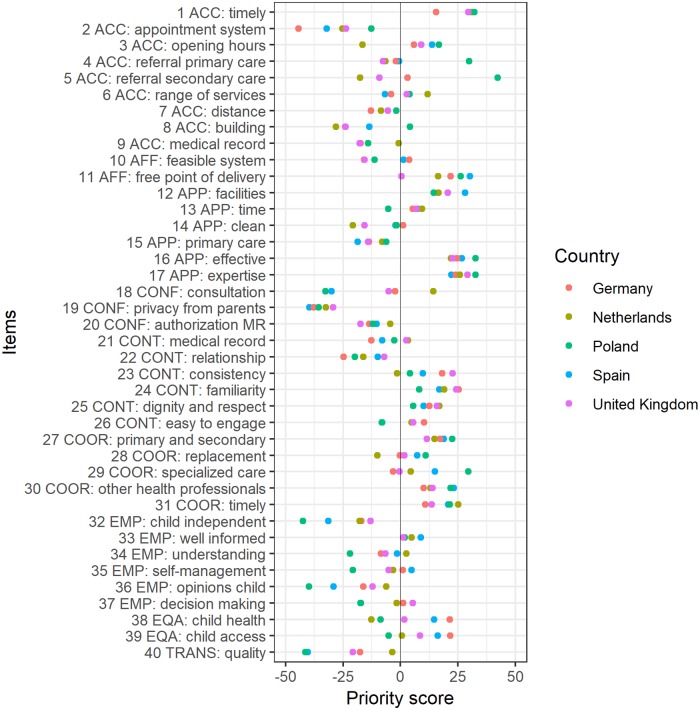
Priority scores per aspect of quality of primary care for children per country.

Priority scores are significantly different between countries. Fig 3 shows the range of priority scores, illustrated by the scores per item per country. Examples of quality aspects which have high overall priority, but which have a difference of more than 30% in priority score between countries are:

- Primary care services for children have ample opening hours, the after-hour care arrangements are good enough, and home-visits are planned if needed (item 3) is relatively important in Poland and Spain, not so important in the Netherlands (F = 26.0; p<0.000).
- Children and/or their parents can make an appointment with secondary or other healthcare providers without a referral from a primary care provider (item 5) is very important in Poland, not so important in the Netherlands and United Kingdom (F = 87.5; p<0.000).
- A child’s health is not influenced by the parents’ social status, economic situation, racial or ethnic background and/or geographic location (item 38) is very important in Germany and Spain, less important in the other countries (F = 25.5; p<0.000).
- Children and/or their parents can make an appointment with other primary care providers without a referral from the main primary care provider (item 4) is very important in Poland, not so important in the other countries (F = 41.2; p<0.000).

### Differences between parents and non-parents

For 11 out of 40 items, parents with children below and above 18 and respondents without children have significantly different perceptions of the quality of primary health care (Pearson’s Chi Square < p = 0.05). The main difference between the three groups is that respondents without children are more often neutral in their opinion compared to parents. There is no consistent difference in opinion between parents with children < 18 and ≥ 18. With regard to satisfaction with care, parents are slightly more satisfied than non-parents, with 68% of parents with children < 18 scoring satisfaction with care with a seven or higher, compared to 63% of parents with children ≥ 18 and 56% of respondents without children (Pearsons Chi-Square p<0.000). See S2 Appendix.

With regard to their priorities, the three groups agree on the importance of eight out of ten items (ranked in the top 10 in all three groups). Health care priorities include timely access (item 1), free at point of delivery (item 11), facilities (item 12), effectiveness (item 16), expertise (item 17), familiarity (item 24), coordination between primary and secondary care (item 27) and timely coordination (item 31). Differences in priorities are found with regard to the following items: child access (item 39) is ranked 7^th^ in non-parents and lower in parents; opening hours are ranked 9^th^ in parents with children < 18 and lower in non-parents and parents of children ≥ 18; and the availability of specialized care within the primary care practice is ranked 8^th^ in parents of children ≥ 18, and somewhat lower by non-parents and parents of children < 18. See S2 Appendix.

### Priority setting for policy makers

**Fig 4:**
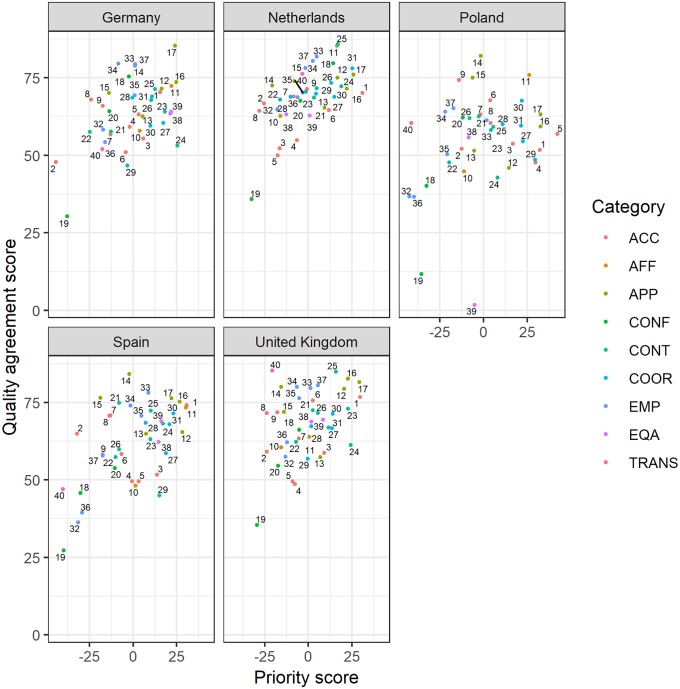
a-e. Priority scores combined with perceived quality agreement scores for each of the 40 quality aspects, given by respondents of the five countries. The colors of the numbers indicate the attribute where the quality aspect belongs to; see S1 Table for the full list of quality aspects, item number and descriptions.

For each country, we combined priority scores with quality agreement scores. See Fig 4a–4e. The aspects with low (perceived) quality and high priority (in the lower left quadrant of the figure) are those with the highest potential for improvement, which are also most important according the respondents from that country. These items differ per country (Table 4).

**Table 4:** Overview of the quality aspects with a high potential for improvement, presented for each of the five countries.

Country	Attribute	Quality aspect (item number)
Germany	Continuous	All healthcare providers involved in the care of a child know about each other’s involvement, trust each other and work well together (item 24).
	Accessible	Primary care services for children have ample opening hours, the after-hour care arrangements are good enough, and home-visits are planned if needed (item 3).
	Coordinated	If the main primary care provider of a child is not able to meet the needs of that child, that care can be given by other health professionals within the primary care practice (item 30).
	Coordinated	If a child needs specialised and long-term care, hospitals and primary care providers collaborate to offer care close to the child’s home (item 27).
	Affordable	The effort needed to get coverage and/or repayment for any out-of-pocket cost of primary care for a child is reasonable and feasible (item 10).
Netherlands	Appropriate	Primary care providers are able to dedicate enough time to working with a child (item 13).
	Accessible	Children and/or their parents know about the range of services available in primary care and how they can access them (item 6).
Poland	Continuous	All healthcare providers involved in the care of a child know about each other’s involvement, trust each other and work well together (item 24).
	Appropriate	In primary care, the facilities and equipment are available to deliver the services that are needed for children (item 12).
	Accessible	Children and/or their parents can make an appointment with other primary care providers without a referral from the main primary care provider (item 4).
	Coordinated	Specialised care (e.g. physiotherapy, dental healthcare, psychological care, specialised chronic care nurses) is available to a child within the primary care provider’s practice (item 29).
	Accessible	Primary care providers provide care within a reasonable amount of time, given the severity of the health issue (item 1).
Spain	Accessible	Primary care services for children have ample opening hours, the after-hour care arrangements are good enough, and home-visits are planned if needed (item 3).
	Coordinated	Specialised care (e.g. physiotherapy, dental healthcare, psychological care, specialised chronic care nurses) is available to a child within the primary care provider’s practice (item 29).
United Kingdom	Continuous	All healthcare providers involved in the care of a child know about each other’s involvement, trust each other and work well together (item 24).
	Accessible	Primary care services for children have ample opening hours, the after-hour care arrangements are good enough, and home-visits are planned if needed (item 3).
	Appropriate	Primary care providers are able to dedicate enough time to working with a child (item 13).

## Discussion

The goal of this study was to elicit formative values from the citizens in five European countries and to determine citizen priorities in the assessment of the quality of a child-oriented primary care system as adopted in the MOCHA working model.

### Perceived quality and priorities

The results indicate that there are significant differences between countries in overall satisfaction with primary care for children, citizen perception of the quality of specific aspects of the primary care system and citizen priorities with regard to what aspects of care are important when judging the quality of the primary care system.

The most important priorities within the five countries are related to aspects of accessibility (timeliness), appropriateness (skills/competences, management, facilities), affordability (no costs), continuity (informational, dignity/respect), and coordination (swift referrals, collaboration). Priorities can be difficult to interpret, because it is hard to separate whether something is a priority because of current poor performance of the system, or because the prioritized aspect of care represents a core belief of what primary care for children should entail. When we combine priorities with perceived quality of the health care system, the results of this study indicate that high priorities are not equal to low perceived quality, suggesting that a basic belief of what is important, independent from current performance of the health care system, is present in our results.

An important aspect with regard to quality vs. priority is the aspect of child autonomy. An earlier study within the MOCHA project indicated that, based on the experiences of parents of children aged under 18 years, improvements with regard to the autonomy aspects of healthcare services are possible in the five countries [29]. The results of our study indicate that citizens in Poland and Spain judge autonomy, empowerment and confidentiality to be low in their country. However, some of these aspects, such as whether a child can limit their parents’ access to the child’s medical records, and whether a child can express his opinions about his health management independently from his parents, are also considered of lesser priority.

Our findings on citizen’s experiences are difficult to compare with other studies. Patient-Reported Experience Measures (PREMs) are increasingly being used in the evaluation of quality of child health care. One of the MOCHA studies summarized, based on results of a literature review and a survey among the 30 European country agents, that Croatia, Denmark and Germany can be identified as early adopters of the use of these measures in the evaluation of quality of child health care. Along with these, England and Ireland have showed a more mature propensity towards their use [30]. However, these countries’ reports could not be used to compare results either because they were not retrievable or in the countries’ own language, or they were disease- or domain-specific (e.g. on diabetes, or hospital care). With regard to priority setting, the study that is closest to our study is the review of Kleij et al. [8]. This review found that process attributes, e.g., care provider, shared decision making, waiting time, information provision, etc., were most often the ones of highest importance for participants in the studies included in the review.

Although in our study there are some aspects of care which seem to be universally prioritized, each country also has its very specific priorities. For instance, it is most important to respondents in Poland that children and/or their parents can make an appointment with other primary or secondary healthcare providers without a referral from a primary care provider, indicating the value of a current characteristic of healthcare. This was prioritized much lower in other countries, which currently do not have that option.

### Potential for improvement

Some aspects of quality of primary care are universally judged low, but are not a priority to the citizens in all countries. An example of such an item is whether a child can express his opinions about his health management independently from his parents (empowerment). If these findings were replicated in all countries, it could be that this is not a determinant of a high-quality primary care system according to the citizen, which is often the payer of the health care system. However, it is also very likely that parents, or even children, have different views on the priority of autonomy in (primary) health care. This is supported by the findings of a qualitative study of Alma et al. [31] with interviews of children indicating that communication and relationships with health care professionals, being part of the conversations, and being involved in managing their own care, are things that need to be improved in primary care.

The results of our study indicate that strengths and weaknesses of the current primary care system and the citizens’ priorities differ per country. This means that the potential for improvement is different in each country. A major yield of this study is that by combining priority scores with an evaluation of the perceived quality for each of the five countries, it became possible to identify the most important areas of potential improvement in each country.

A next step could be to analyse whether strengths in one country can be transferred to another country. For example, an interesting quality aspect with respect to transferability [32] is the item: All healthcare providers involved in the care of a child know about each other’s involvement, trust each other and work well together. This item on continuity of care had the highest priority to respondents from Germany (paediatrician-led system and open access), and the quality of the current system was judged low on this item by German respondents. The quality of the Spanish (paediatrician-led system with primary care as gatekeeper) and Dutch (GP-led system with primary care as gatekeeper) primary care systems are judged relatively good. It would be interesting to study how the Dutch, Spanish and German systems promote coordination between healthcare providers, and whether aspects of the Dutch or Spanish system with respect to this item could be transferred to Germany.

### Strengths and limitations

A strength of this study is that the best-worst scaling methodology was used to prioritize aspects of quality of care based on their importance for a high-quality primary care system for children. Best-worst scaling forces respondents to prioritize, in contrast to more traditional verbal or numerical rating scales per item. Even with the high number of items to prioritize, and thus the relatively low number of times each item was shown to keep the survey burden acceptable, this method proved to be successful in eliciting the citizen’s priorities and allowed for discrimination of priorities between the five countries.

A limitation of this study is that the samples of the United Kingdom, the Netherlands, Spain and Poland show an overrepresentation of middle and/or high-educated respondents and the sample of Germany an overrepresentation of low-educated respondents. The extent to which the results of the study are influenced by educational level is not known. Unfortunately, the difference between educational systems of countries does not permit to repeat the analyses for the high, middle, and low educational groups in the merged sample and the sample size per country does not allow sub-analyses according to educational level (or other background characteristics) per country.

Respondents in this sample were recruited through the internet. This has limited the participation of non-internet using citizens to the questionnaire, and limits the generalizability of the results to the country population level. Future research should focus on distributing the questionnaire through other channels, which are also accessible to less digitalized citizens of the countries.

In order to include respondents of countries with a diversity of primary care systems, the criteria of ‘lead-practitioner’, ‘gatekeeping’ and ‘organizational place of preventive services’ were chosen. Of course, using only these criteria as basis for distinguishing models is an oversimplification of the necessary criteria for appraisal. The design of our study does not allow us to relate differences in perceived quality of or priorities for attributes of the current system to specific underlying system differences, like the criterion ‘lead practitioner’. Therefore, our results cannot easily be generalised to other countries, even not to those with comparable healthcare systems, but should be interpreted within the country’s own context.

### Recommendations

According to the principles of the WHO people should have a say in the planning of health priorities and in how these priorities are implemented in their community [33]. This study shows that the POCHA questionnaire can be used to elicit perceived quality and priorities of citizens with regard to the quality of the current primary care system for children in their country. If prioritization by citizens is combined with perceived quality evaluation, the results of such an exercise may help direct efforts of policy makers for improving the child healthcare system.

Citizen experiences and priorities have been shown to be important to estimate whether strengths of primary care delivery in one country can be transferred to another country [32]. Policy makers of countries that participated in our study that plan healthcare reform, can use the results of this survey when they want to take into account the citizen’s perspective in their decision process. Other countries can use the POCHA questionnaire to assess citizen experiences and priorities in their own country.

Preferably, the questionnaire should first be further refined, shortened and validated, before it is used in other countries. In addition, it would be interesting to develop a child-specific variant of the POCHA questionnaire with children and adolescents, as this may uncover other priorities for primary care.

## Supporting information

S1 Table List of quality attributes, quality aspects (items) and descriptions. (DOCX)

S2 Table Comparison of the percentage of agreement (summed percentage of respondents that agree and strongly agree) with the statements on each of the 40 attribute-items of quality of the primary care system, indicated by the respondents of the 5 countries. (DOCX)

S1 Appendix Respondent dropout after recruitment. (DOCX)

S2 Appendix Subgroup analysis (extended results). (DOCX)

S1 Minimal Dataset POCHA SPSS file. (SAV)
