# Supplementary material for: Primary care in five European countries: A citizens’ perspective on the quality of care for children
Source: PLoS One. 2019 Nov 11;14(11):e0224550. doi: 10.1371/journal.pone.0224550 (PMC6844459; doi:10.1371/journal.pone.0224550)
Supplement: S1 Table — (DOCX) [file pone.0224550.s001.docx]

## S1 Table. List of quality attributes, quality aspects (items) and descriptions

| attribute | aspect | Item Description |
| --- | --- | --- |
| accessible | ACC; timely  (item 1) | Primary care providers provide care within a reasonable amount of time, given the severity of the health issue |
|  | ACC; appointment system  (item 2) | Primary care services for children have a convenient appointment system |
|  | ACC; opening hours  (item 3) | Primary care services for children have ample opening hours, the after-hour care arrangements are good enough, and home-visits are planned if needed |
|  | ACC; referral primary care  (item 4) | Children and/or their parents can make an appointment with other primary care providers without a referral from the main primary care provider |
|  | ACC; referral secondary care  (item 5) | Children and/or their parents can make an appointment with secondary or other health care providers without a referral from a primary care provider |
|  | ACC; range of services  (item 6) | Children and/or their parents know about the range of services available in primary care and how they can access them |
|  | ACC; distance  (item 7) | Primary care services for children are nearby, and are easily reached on foot or by bike, car and/or public transport |
|  | ACC; building  (item 8) | Primary care services for children can be easily accessed with buggies, wheelchairs, or other assistive devices |
|  | ACC; medical record  (item 9) | A child and/or his parents have access to a child's medical records |
| affordable | AFF; feasible system  (item 10) | The effort needed to get coverage and/or repayment for any out-of-pocket cost of primary care for a child is reasonable and feasible |
|  | AFF; free point of delivery  (item 11) | Primary care services for a child are free at the point of delivery, or out-of-pocket costs are fully covered or repaid by insurance |
| appropriate | APP; facilities  (item 12) | In primary care, the facilities and equipment are available to deliver the services that are needed for children |
|  | APP; time  (item 13) | Primary care providers are able to dedicate enough time to working with a child |
|  | APP; clean  (item 14) | Primary care services for children are provided in a clean and appealing setting |
|  | APP; primary care  (item 15) | Health care for children is provided in the primary care setting whenever possible |
|  | APP; effective  (item 16) | In primary care, a child’s health problems are effectively managed |
|  | APP; expertise  (item 17) | Primary care providers have the skills and competences to provide the care a child needs |
| confidential | CONF; consultation  (item 18) | A child has the right to a confidential consultation with the primary care provider |
|  | CONF; privacy from parents  (item 19) | In primary care, a child can limit their parents’ access to the child’s medical records in order to protect his privacy |
|  | CONF; authorization MR  (item 20) | In primary care, the child and/or the parents have to authorise other health care providers accessing the child’s medical records |
| continuous | CONT; medical record  (item 21) | Any primary care provider caring for a child has access to a full overview of that child’s medical records |
|  | CONT; relationship  (item 22) | A child and his parents have a long-term relationship with primary care providers, beyond specific episodes of illness or disease |
|  | CONT; consistency  (item 23) | Primary care providers offer a consistent and coherent approach to the management of a child's health, which is adjusted when the needs of that child change |
|  | CONT; familiarity  (item 24) | All health care providers involved in the care of a child know about each other’s involvement, trust each other and work well together |
|  | CONT; dignity and respect  (item 25) | Primary care providers treat children and their parents with dignity and respect |
|  | CONT; easy to engage  (item 26) | Primary care providers are easy to engage, considerate and non-judgmental of parents and children |
| coordinated | COOR; primary and secondary  (item 27) | If a child needs specialised and long-term care, hospitals and primary care providers collaborate to offer care close to the child's home |
|  | COOR; replacement  (item 28) | If a child’s main primary care provider is sick or on leave, a replacement is available quickly |
|  | COOR; specialized care  (item 29) | Specialised care (e.g. physiotherapy, dental health, psychological care, specialised chronic care nurses) is available to a child within the primary care provider’s practice |
|  | COOR; other health professionals  (item 30) | If the main primary care provider of a child is not able to meet the needs of that child, that care can be given by other health professionals within the primary care practice |
|  | COOR; timely  (item 31) | In primary care, a child is referred to other health care providers swiftly if this is needed |
| empowering | EMP; child independent  (item 32) | In primary care, a child can express his opinions about his health management independently from his parents |
|  | EMP; well informed  (item 33) | In primary care, children and their parents are well informed about (the management of) the child’s health |
|  | EMP; understanding  (item 34) | Primary care providers make reasonable efforts to ensure that a child and his parents understand the information they provide |
|  | EMP; self-management  (item 35) | Children and/or their parents are assisted by primary care providers in acquiring the skills to promote and manage the child’s health |
|  | EMP; opinions child  (item 36) | Primary health care providers take into account the child’s opinion on their management of the child’s health |
|  | EMP; decision-making  (item 37) | In primary care, children and/or their parents are involved in decisions about the management of the child’s health |
| equable | EQA; child health  (item 38) | A child’s health is not influenced by the parents’ social status, economic situation, racial or ethnic background and/or geographic location |
|  | EQA; child access  (item 39) | A child’s access to primary care and the quality of care he receives are not influenced by the parents’ social status, economic situation, racial or ethnic background and/or geographic location |
| transparent | TRANS; quality  (item 40) | Primary care providers are open about the quality of the health services available to children |
