# Supplementary material for: Primary care in five European countries: A citizens’ perspective on the quality of care for children
Source: PLoS One. 2019 Nov 11;14(11):e0224550. doi: 10.1371/journal.pone.0224550 (PMC6844459; doi:10.1371/journal.pone.0224550)
Supplement: S2 Table — (DOCX) [file pone.0224550.s002.docx]

## S2 Table. Comparison of the percentage of agreement (summed percentage of respondents that agree and strongly agree) with the statements on each of the 40 attribute-items of quality of the primary care system, indicated by the respondents of the 5 countries.

##

| **Attribute-item** | **% Agreement with statement** | | | | | | | |
| --- | --- | --- | --- | --- | --- | --- | --- | --- |
|  | GE | NL | POL | ES | UK | ALL | Chi^2^ | Sign. |
| ACC; timely (item 1) | 70 | 70 | 52 | 74 | 77 | 69 | 22.2 | 0.000 |
| ACC; appointment system (item 2) | 48 | 67 | 52 | 65 | 59 | 58 | 17.7 | 0.001 |
| ACC; opening hours (item 3) | 55 | 52 | 54 | 52 | 59 | 54 | 5.7 | 0.227 |
| ACC; referral primary care (item 4) | 59 | 55 | 48 | 50 | 49 | 52 | 10.2 | 0.037 |
| ACC; referral secondary care (item 5) | 63 | 50 | 57 | 50 | 50 | 54 | 7.0 | 0.134 |
| ACC; range of services (item 6) | 51 | 65 | 68 | 58 | 76 | 63 | 16.4 | 0.003 |
| ACC; distance (item 7) | 57 | 69 | 63 | 71 | 63 | 65 | 6.0 | 0.196 |
| ACC; building (item 8) | 68 | 64 | 60 | 71 | 72 | 67 | 12.6 | 0.014 |
| ACC; medical record (item 9) | 66 | 71 | 74 | 58 | 72 | 68 | 5.9 | 0.204 |
| AFF; feasible system (item 10) | 58 | 63 | 45 | 48 | 60 | 55 | 15.0 | 0.005 |
| AFF; free point of delivery (item 11) | 72 | 85 | 76 | 73 | 64 | 74 | 7.1 | 0.130 |
| APP; facilities (item 12) | 72 | 75 | 46 | 65 | 79 | 67 | 52.0 | 0.000 |
| APP; time (item 13) | 63 | 65 | 51 | 65 | 57 | 60 | 9.3 | 0.054 |
| APP; clean (item 14) | 79 | 73 | 82 | 84 | 80 | 80 | 8.8 | 0.067 |
| APP; primary care (item 15) | 70 | 74 | 75 | 76 | 72 | 73 | 2.4 | 0.660 |
| APP; effective (item 16) | 74 | 72 | 59 | 75 | 83 | 72 | 21.1 | 0.000 |
| APP; expertise (item 17) | 85 | 76 | 63 | 76 | 82 | 76 | 30.0 | 0.000 |
| CONF; consultation (item 18) | 75 | 80 | 40 | 46 | 66 | 61 | 74.3 | 0.000 |
| CONF; privacy from parents (item 19) | 30 | 36 | 12 | 27 | 35 | 28 | 45.6 | 0.000 |
| CONF; authorization MR (item 20) | 64 | 68 | 62 | 54 | 55 | 60 | 7.2 | 0.126 |
| CONT; medical record (item 21) | 58 | 69 | 63 | 75 | 73 | 67 | 15.5 | 0.004 |
| CONT; relationship (item 22) | 58 | 68 | 48 | 57 | 62 | 59 | 18.7 | 0.001 |
| CONT; consistency (item 23) | 64 | 71 | 58 | 63 | 73 | 66 | 8.9 | 0.064 |
| CONT; familiarity (item 24) | 53 | 72 | 43 | 68 | 61 | 60 | 29.5 | 0.000 |
| CONT; dignity and respect (item 25) | 71 | 86 | 59 | 72 | 85 | 75 | 22.9 | 0.000 |
| CONT; easy to engage (item 26) | 68 | 72 | 62 | 60 | 72 | 67 | 7.8 | 0.097 |
| COOR; primary and secondary (item 27) | 60 | 69 | 55 | 59 | 67 | 62 | 17.1 | 0.002 |
| COOR; replacement (item 28) | 69 | 69 | 60 | 68 | 67 | 67 | 10.3 | 0.036 |
| COOR; specialized care (item 29) | 47 | 70 | 49 | 45 | 57 | 53 | 24.1 | 0.000 |
| COOR; other health professionals (item 30) | 60 | 73 | 68 | 71 | 71 | 69 | 5.3 | 0.259 |
| COOR; timely (item 31) | 69 | 78 | 60 | 68 | 67 | 68 | 14.5 | 0.006 |
| EMP; child independent (item 32) | 58 | 65 | 37 | 36 | 57 | 51 | 45.3 | 0.000 |
| EMP; well informed (item 33) | 79 | 82 | 61 | 78 | 80 | 76 | 18.6 | 0.001 |
| EMP; understanding (item 34) | 80 | 80 | 64 | 74 | 80 | 76 | 16.9 | 0.002 |
| EMP; self-management (item 35) | 69 | 70 | 50 | 71 | 76 | 67 | 28.2 | 0.000 |
| EMP; opinions child (item 36) | 54 | 69 | 37 | 39 | 62 | 52 | 40.3 | 0.000 |
| EMP; decision-making (item 37) | 79 | 78 | 65 | 58 | 80 | 72 | 24.5 | 0.000 |
| EQA; child health (item 38) | 63 | 63 | 56 | 62 | 69 | 63 | 5.3 | 0.253 |
| EQA; child access (item 39) | 63 | 69 | 69 | 64 | 74 | 68 | 1.7 | 0.784 |
| TRANS; quality (item 40) | 76 | 85 | 47 | 52 | 65 | 65 | 60.4 | 0.000 |
