# Supplementary material for: Primary care in five European countries: A citizens’ perspective on the quality of care for children
Source: PLoS One. 2019 Nov 11;14(11):e0224550. doi: 10.1371/journal.pone.0224550 (PMC6844459; doi:10.1371/journal.pone.0224550)
Supplement: S1 Appendix — (DOCX) [file pone.0224550.s003.docx]

**S1 Appendix: Respondent dropout after recruitment**

| Respondent Sample | Recruitment | Excluded | Dropout % | Valid Respondents |
| --- | --- | --- | --- | --- |
| UK | 530 | 34 | 6.4 | 496 |
| NL | 526 | 57 | 10.8 | 469 |
| DE | 526 | 57 | 10.8 | 469 |
| ES | 528 | 37 | 7.0 | 491 |
| PL | 530 | 52 | 9.8 | 478 |
| Total | 2640 | 237 | 9.0 | 2403 |
