# Supplementary material for: Primary care in five European countries: A citizens’ perspective on the quality of care for children
Source: PLoS One. 2019 Nov 11;14(11):e0224550. doi: 10.1371/journal.pone.0224550 (PMC6844459; doi:10.1371/journal.pone.0224550)
Supplement: S2 Appendix — (DOCX) [file pone.0224550.s004.docx]

**S2 Appendix: Subgroup Analysis (extended results)**

We divided the whole sample in three groups:

- Parents of children < 18 (n=872)
- Parents of children ≥ 18 (n=559)
- Non-parents (n=972)

**Perceptions**

For 11 out of 40 items, parents with children below and above 18 and respondents without children have significantly different perceptions (Pearson’s Chi Square < p = 0.05)

Parents with children < 18, parents with children ≥ 18 and respondents without children differ in their perception whether:

1. Children and/or their parents can make an appointment with other primary care providers without a referral from the main primary care provider; respondents without children more neutral, less positive. ACC; referral primary care (item 4)
2. Children and/or their parents know about the range of services available in primary care and how they can access them; respondents without children more negative and neutral. ACC; range of services (item 6)
3. Primary care services for children are nearby, and are easily reached on foot or by bike, car and/or public transport; respondents without children more neutral. ACC; distance (item 7)
4. Primary care services for a child are free at the point of delivery, or out-of-pocket costs are fully covered or repaid by insurance; respondents without children more neutral. AFF; free point of delivery (item 11)
5. Primary care services for children are provided in a clean and appealing setting; parents with children < 18 more negative. APP; clean (item 14)
6. Primary care providers have the skills and competences to provide the care a child needs; respondents without children more neutral. APP; expertise (item 17)
7. Any primary care provider caring for a child has access to a full overview of that child's medical records; respondents without children more neutral. CONT; medical record (item 21)
8. A child and his parents have a long-term relationship with primary care providers, beyond specific episodes of illness or disease; respondents without children more neutral. CONT; relationship (item 22)
9. Primary care providers treat children and their parents with dignity and respect; respondents without children more neutral. CONT; dignity and respect (item 25)
10. Primary care providers are easy to engage, considerate and non-judgmental of parents and children; respondents without children more neutral. CONT; easy to engage (item 26)
11. If a child's main primary care provider is sick or on leave, a replacement is available quickly; respondents without children more neutral. COOR; replacement (item 28)

The main difference between groups is that respondents without children are more often neutral in their opinion compared to parents. There is no consistent difference in opinion between parents with children below and above 18.

**Satisfaction with primary health care for children**

With regard to satisfaction with care, parents are more slightly more satisfied than non-parents, with 68% of parents with children < 18 scoring satisfaction with care with a seven or higher, compared to 63% of parents with children ≥ 18 and 56% of respondents without children (Pearsons Chi-Square p<0.000)

**Priorities**

With regard to priorities the groups agree on the importance of eight out of ten items (ranked in the top 10 in all three groups)

- Primary care providers provide care within a reasonable amount of time, given the severity of the health issue. ACC; timely (item 1)
- Primary care services for a child are free at the point of delivery, or out-of-pocket costs are fully covered or repaid by insurance. AFF; free point of delivery (item 11)
- In primary care, the facilities and equipment are available to deliver the services that are needed for children. APP; facilities (item 12)
- In primary care, a child’s health problems are effectively managed. APP; effective (item 16)
- Primary care providers have the skills and competences to provide the care a child needs. APP; expertise (item 17)
- All health care providers involved in the care of a child know about each other’s involvement, trust each other and work well together. CONT; familiarity (item 24)
- If a child needs specialised and long-term care, hospitals and primary care providers collaborate to offer care close to the child's home. COOR; primary and secondary (item 27)
- In primary care, a child is referred to other health care providers swiftly if this is needed. COOR; timely (item 31)

Disagreement between groups with regard to priorities (above average difference in ranking, ranked in top 10 by one of the groups) were the following:

- A child’s access to primary care and the quality of care he receives are not influenced by the parents’ social status, economic situation, racial or ethnic background and/or geographic location. This item is ranked 7^th^ in respondents without children, 13^th^ in parents with children ≥ 18 and 19^th^ in parents with children < 18. EQA; child access (item 39)
- Primary care services for children have ample opening hours, the after-hour care arrangements are good enough, and home-visits are planned if needed. This item is ranked 9^th^ in respondents in parents with children < 18, 18^th^ and 20^th^ in respondents without children and with children < 18. ACC; opening hours (item 3)
- Specialised care (e.g. physiotherapy, dental health, psychological care, specialised chronic care nurses) is available to a child within the primary care provider’s practice. This item is ranked 8^th^ in respondents with children ≥ 18, 13^th^ by parents with children < 18, 14^th^ in respondents without children. COOR; specialized care (item 29)
